# Sperm Incubation in Biggers–Whitten–Whittingham Medium Induces Capacitation-Related Changes in the Lizard *Sceloporus torquatus*

**DOI:** 10.3390/ani14091388

**Published:** 2024-05-06

**Authors:** Uriel Ángel Sánchez-Rivera, Norma Berenice Cruz-Cano, Alfredo Medrano, Carmen Álvarez-Rodríguez, Martín Martínez-Torres

**Affiliations:** 1 Laboratorio de Biología de la Reproducción, Facultad de Estudios Superiores Iztacala, Universidad Nacional Autónoma de México, Mexico City 54090, Mexico; bionormacruz.7@gmail.com (N.B.C.-C.);; 2 Laboratorio de Reproducción, Facultad de Estudios Superiores Cuautitlán, Universidad Nacional Autónoma de México, Mexico City 54714, Mexico; amedrano@unam.mx; 3 Posgrado en Ciencias de la Producción y de la Salud Animal, Universidad Nacional Autónoma de México, Mexico City 04510, Mexico

**Keywords:** sperm capacitation, BWW medium, lizard, incubation, assisted reproduction techniques

## Abstract

**Simple Summary:**

Sperm acquire the ability to fertilize the egg during their transit through the female reproductive tract. This process, known as sperm capacitation, is well recognized in mammals and can be accomplished under laboratory conditions using specialized media. However, it remains unknown whether this process occurs in lizards. In this study, we investigated sperm incubation under conditions that promote capacitation to determine if similar changes occurred in their sperm. Our sperm assessment revealed functional changes, such as modifications in movement and staining patterns, commonly observed in mammals. This suggests that sperm capacitation may occur in this group of animals. Understanding sperm physiology is crucial for developing assisted reproduction technologies to aid conservation efforts for threatened species.

**Abstract:**

Sperm capacitation involves biochemical and physiological changes that enable sperm to fertilize the oocyte. It can be induced in vitro under controlled conditions that simulate the environment of the oviduct. While extensively studied in mammals, its approach in lizards remains absent. Understanding the mechanisms that ensure reproduction is essential for advancing the implementation of assisted reproductive technologies in this group. We aimed to perform a sperm analysis to determine if capacitation-related changes were induced after incubation with capacitating media. Fifteen males of *Sceloporus torquatus* were collected during the early stage of the reproductive season. The sperm were isolated from the seminal plasma and then diluted up to a volume of 150 μL using BWW medium to incubate with 5% CO_2_ at 30 °C for a maximum duration of 3 h. A fraction was retrieved hourly for ongoing sperm assessment. The sperm analysis included assessments of its motility, viability, the capacitation status using the chlortetracycline (CTC) assay, and the acrosome integrity with the lectin binding assay to detect changes during incubation. We found that total motility was maintained up to 2 h post incubation, after which it decreased. However, sperm viability remained constant. From that moment on, we observed a transition to a deeper and less symmetrical flagellar bending in many spermatozoa. The CTC assay indicated a reduction in the percentage of sperm showing the full (F) pattern and an increase in those exhibiting the capacitated (B) and reactive (RA) patterns, accompanied by an elevation in the percentage of damaged acrosomes as revealed by the lectin binding assay. In mammals, these changes are often associated with sperm capacitation. Our observations support the notion that this process may also occur in saurian. While sperm analysis is a valuable method for assessing certain functional changes, additional approaches are required to validate this process.

## 1. Introduction

Ejaculated mammalian sperm are morphologically mature but functionally unable to fertilize [1]. Sperm undergo biochemical and physiological changes to become capable of binding and interacting with the oocyte. These modifications include cholesterol efflux, increased membrane fluidity, changes in intracellular ion concentrations, pH elevation, alterations in protein kinase activity, and tyrosine phosphorylation, and occur during their passage through the female reproductive tract in a complex process known as sperm capacitation [2,3,4]. This process can be accomplished in vitro under controlled conditions by recreating the oviductal environment using defined media supplemented with essential ions such as bicarbonate, calcium, albumin, and energy substrates [5]. 

Although fully recognized in mammals, sperm capacitation remains uncertain in lizards, despite sharing characteristics such as internal fertilization and the possession of the epididymis, where sperm acquire motility [6]. Females also have the ability to store sperm in the oviducts, allowing an asynchrony between mating and ovulation [7,8]. These characteristics suggest that spermatozoa may require physiological changes after insemination to acquire fertilization ability. Thus far, only one study has conclusively shown sperm capacitation in crocodiles by noting increased intracellular levels of cyclic adenosine monophosphate (cAMP), which enhance motility and elevate protein phosphorylation levels [9]. Moreover, epididymal spermatozoa in *Lacerta vivipara* exhibit increased motility when incubated in a medium containing caffeine, a phosphodiesterase inhibitor known to elevate cAMP levels [10]. These observations suggest that this mechanism may indeed occur within lizards.

Given the ongoing global decline in herpetofauna [11,12], the comprehension of reproductive biology is crucial for the development of any assisted reproductive technologies (ARTs) [13]. We selected *Sceloporus torquatus* as a model for advancing ARTs due to our understanding of its reproductive biology [14,15]. We developed non-invasive semen collection methods by establishing the time to obtain greater volumes and generated sperm quality references [16,17]. However, our efforts in sperm cryopreservation have yielded a low success rate in post-thawing recovery [18]. The above highlights that it is crucial to grasp semen quality parameters, prevent spontaneous acrosome reactions [5], and enhance post-procedural recovery [19]. Moreover, artificial insemination has been unsuccessful (unpublished data), possibly due to inadequate manipulation and preparation of both gametes [20]. These challenges underscore the importance of studying sperm physiology for successful ART implementation. In order to fill the gaps in knowledge regarding sperm capacitation, we conducted an incubation study using Biggers–Whitten–Whittingham (BWW) medium. Our aim was to determine by means of sperm analysis if functional changes similar to those found in mammals were induced.

## 2. Materials and Methods

### 2.1. Animals

The capture of 15 adult males of *Sceloporus torquatus* (SVL > 70 mm) [21] was conducted in the Sierra de Guadalupe State Park, Coacalco, State of Mexico (19°61′ N, 99°11′ W, 2480 m altitude), under scientific collection licenses SPA/DGVD/086681/21 and SPARN/DGVD/12218/23 granted by the Secretaría del Medio Ambiente y Recursos Naturales. The collection occurred during the early stage of the reproductive season (October–November) in both 2021 and 2022. Morphometric data of the animals were recorded, including snout–vent length (using digital Vernier calipers to the nearest 0.01 mm) and body weight for each individual (measured using a digital scale with 0.1 g precision). The lizards were kept in outdoor enclosures measuring 3.0 × 5.0 × 2.0 m, with access to food and water, and then released into their natural habitat after completing experimental procedures.

### 2.2. Semen Collection and Incubation

Semen was collected by gently pressing the genital papillae, following the method described by Martínez-Torres et al. [16]. We registered the number of ejaculates, the total semen volume, and sperm concentration for each male. The ejaculates were washed with PBS and isolated from seminal plasma via double centrifugation at 978× *g*-force for 10 min at room temperature. The sperm samples were diluted to a final volume of 150 μL using BWW medium, with the following composition: 120 mM NaCl, 4.6 mM KCl, 1.7 mM CaCl_2_·2H_2_O, 1.2 mM KH_2_PO_4_, 1.2 mM MgSO_4_·7H_2_O, 5.6 mM D-glucose, 0.27 mM sodium pyruvate, 44 mM sodium lactate, 5 U/mL penicillin, 5 mg/mL streptomycin, 20 mM HEPES, 3 mg/mL BSA, and 25 mM NaHCO_3_, at a pH of 7.4 and an osmolarity of 300 mOsm [9]. All chemicals were purchased from Sigma. A portion of 30 μL was retrieved for assessment at 0 h, while the remaining sample was incubated with 5% CO_2_ at 30 °C for up to 3 h. Additionally, a fraction was retrieved each hour for ongoing semen assessment.

### 2.3. Sperm Assessment

#### 2.3.1. Sperm Motility

To assess sperm motility, 5 µL of diluted sperm was placed on a slide and viewed under an optical microscope (Leica DM100) at 40× magnification. The percentage of active sperm was determined based on the presence of symmetrical flagellar movement, which propels them in nearly linear progressive trajectories, leading them out of the visual field [16]. It is crucial to differentiate between actively moving sperm and those passively carried away by the medium flow. 

#### 2.3.2. Sperm Viability

**Figure 1:**
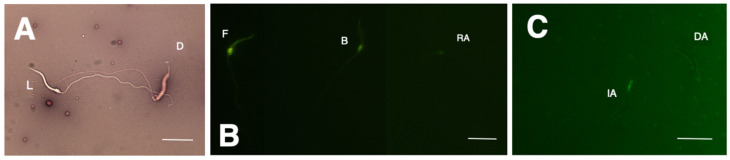
Representative images of *Sceloporus torquatus* sperm evaluation. (**A**) shows live (L) and dead (D) spermatozoa stained with eosin nigrosin, (**B**) shows full (F), band (B), and acrosome-reacted (RA) patterns indicative of capacitance state, as revealed by the CTC assay, and (**C**) shows sperm with intact (IA) and damaged acrosome (DA) spermatozoa, as revealed by the lectin binding assay. Linear bars correspond to 10 µm.

To perform a sperm viability test, 5 µL of diluted sperm was taken and mixed with an equal volume of eosin-nigrosin dye on a slide [18]. The mixture was allowed to dry and then observed under an optical microscope at 100× magnification. Any unstained sperm was considered live, while sperm stained in dark pink was considered dead (Figure 1A). 

#### 2.3.3. Capacitation Status

We prepared a CTC solution (805 μmol clortetracycline, 20 mM Tris, 130 mM NaCl, and 5 mM L-cysteine at a pH of 7.8) using the method described by Naijian et al. [22]. We mixed this solution in a 1:1 ratio with the same volume of 10 µL of diluted sperm. We stopped the reaction by adding 5 µL of 0.2% glutaraldehyde. Then, we prepared smears and examined them under an epifluorescent microscope at 100× magnification. We determined the sperm capacitation state based on the proportion of spermatozoa exhibiting CTC assay patterns (Figure 1B): full/F (uniform fluorescence head), band/B (post-acrosomal region without fluorescence), and acrosome-reacted/AR (fluorescent-free head or a thin fluorescent band on the equatorial segment). 

#### 2.3.4. Acrosome Integrity

The sperm were placed on a slide and left to dry. The slides were permeabilized by immersion in 96% ethanol. We spread 10 µL of fluorescein-conjugated *Pisum sativum* agglutinin (PSA-FITC) lectin and then incubated for 7 min in the dark. After gently washing, we mounted slides with a drop of an antifade solution (220 mM DABCO diluted in glycerol) [23]. The sample was examined under epifluorescence microscopy at 100× magnification to determine the percentage of cells with well-defined acrosomes (Figure 1C). 

### 2.4. Statistical Analysis

The data are presented as mean ± standard error of the mean. Prior to data analysis, we assessed normality and homogeneity using the Shapiro–Wilk and Bartlett tests, respectively. As our data did not meet the assumptions for parametric statistics, we utilized the Kruskal–Wallis test to identify significant differences among the incubation time periods. Subsequently, we conducted Dunn’s post hoc test to determine if there are differences between incubation times for each sperm assessment. We assessed the effect of sperm incubation using the Wilcoxon test, with T0 as the control for each pair. A *p*-value of less than 0.05 was considered statistically significant. We carried out all the analyses and plots using the R (version 4.3.2) software on iOS.

## 3. Results

According to morphometric values, all males (*n* = 15) were considered adults. We obtained semen showing consistent characteristics typical of an ejaculate [16], including volume and sperm concentration (Table 1).

**Figure 2:**
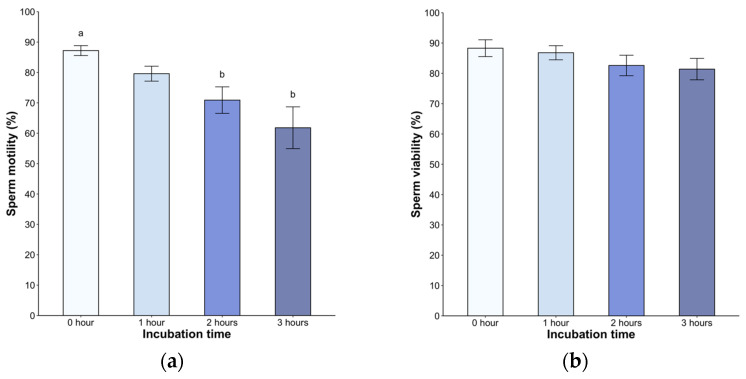
(**a**) Sperm motility and (**b**) viability of *Sceloporus torquatus* incubated in BWW medium. The lines represent the standard error of the mean. Different letters indicate significant differences between incubation times (Dunn, *p* < 0.05).

Following dilution, all samples exhibited high motility, ranging from 79% to 98%. We found a significant decrease in the second (70.9 ± 4.3%) and third hour (61.8 ± 6.8%, *p* < 0.05) post incubation (H = 21.05, *p* < 0.05, Figure 2a). A statistically significant difference was observed in the second and third hour post treatment (*p* < 0.05), but without difference between the hours. Sperm viability remained above 80% throughout the entire 240-min observation period (*p* < 0.05, Figure 2b). Of note, the spermatozoa displayed active linear movement, but a transition to deeper and less symmetrical flagellar bending, with non-linear movement in many spermatozoa, was observed starting at 2 h post incubation.

**Figure 3:**
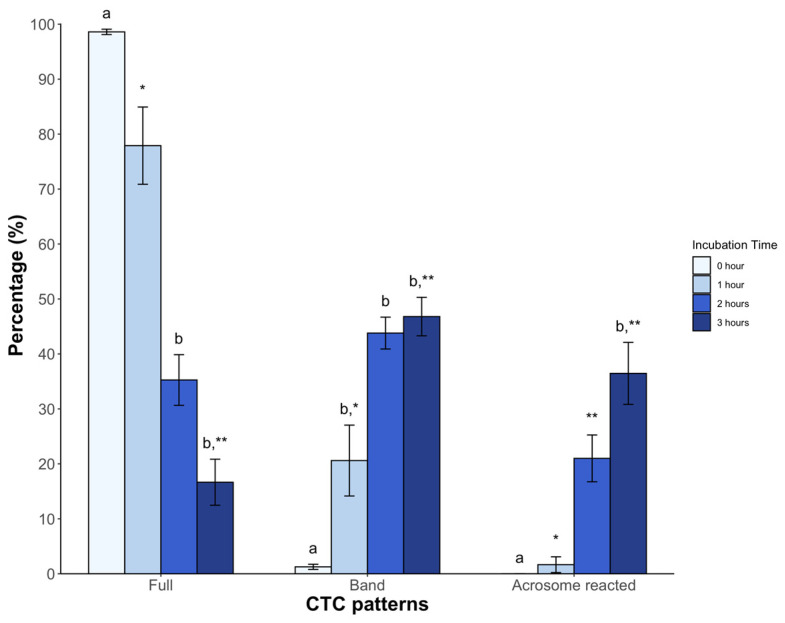
The clorthetracyclin (CTC) sperm patterns of *Sceloporus torquatus* incubated in BWW medium. The lines represent the standard error of the mean. Different letters and asterisks indicate significant differences between incubation times in each pattern (Dunn, *p* < 0.05).

In the case of CTC patterns, a high percentage of sperm showed the full pattern (98.6 ± 0.4%) at time 0, with a significant decrease starting from the second hour (35.2 ± 4.6%) and third hour (16.6 ± 4.1%, *p* < 0.05) post incubation. The band pattern showed low levels immediately after dilution with BWW medium (1.2 ± 0.4%), which gradually increased from the first hour and reached 46.8 ± 3.5% in the third hour. Regarding the acrosome-reacted pattern, it was initially absent but appeared from the second hour of incubation, reaching an average value of 36.4 ± 5.6% (*p* < 0.05) at 3 h (Figure 3).

**Figure 4:**
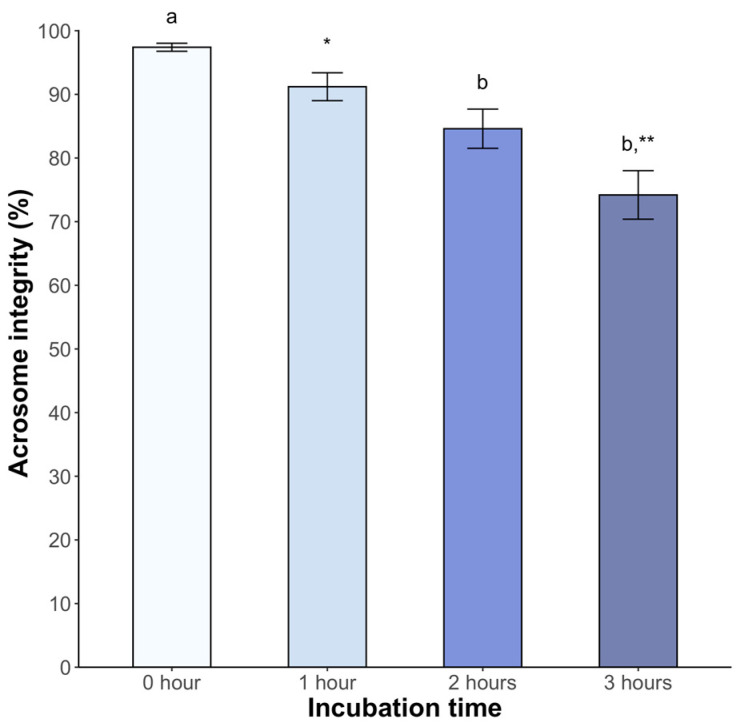
Sperm acrosome integrity of *Sceloporus torquatus* incubated in BWW medium. The lines represent the standard error of the mean. Different letters and asterisks indicate significant differences between incubation times (Dunn, *p* < 0.05).

Significant changes in acrosome integrity were observed. At 2 h post incubation, 84.6 ± 3.1% of spermatozoa maintained integrity (*p* < 0.05). This percentage decreased to 74.2 ± 3.8% at 3 h post incubation (*p* < 0.05) (Figure 4).

## 4. Discussion

Mammalian sperm incubation in defined media reveals biochemical and physiological changes during capacitation [3,24]. Considering the lack of studies about sperm physiology in lizards, it is essential to determine if their sperm undergoes capacitation (as reported in crocodiles) [9] to advance the successful implementation of ARTs in this group. To address this, we incubated *Sceloporus torquatus* spermatozoa in BWW medium at 30 °C with 5% CO_2_ for up to 3 h to assess functional capacitation-related changes in sperm quality.

We observed a consistently high percentage of motility (above 79%), which decreased over time starting at 2 h. This trend suggests that the medium may favor the metabolic processes of sperm [25], potentially because its composition improves cell longevity [26]. Considering the specific variations of each species, the choice of medium is crucial for an adequate manipulation of gametes [27]. BWW medium promotes sperm motility and consistently induces increased cAMP levels and protein tyrosine phosphorylation in mammals [28]. Additionally, it enhanced motility in *Crocodylus porosus* in a 120-min incubation [9]. In light of the observed effect of phosphodiesterase inhibitor (which increases cAMP levels) on *Lacerta vivipara* sperm, resulting in increased motility [10], we hypothesize that this mechanism is conserved in this group and *S. torquatus* would react similarly under capacitation conditions. 

We also noted modifications in motility patterns in many sperm, with increased and deeper flagellar beat amplitude, less symmetrical bending, and nonlinear movement. These observations suggest the hyperactive movement, which may facilitate the zona pellucida penetration during fertilization, as observed in mammals [29]. However, we did not quantify the proportion of spermatozoa undergoing these changes. A comprehensive assessment of motility using computer-assisted sperm analysis (CASA) is essential to detect movement types and evaluate the proportion of hyperactivated sperm [30].

Evaluating the response of sperm metabolism can be challenging due to the complexity of the involved molecules. However, CTC binds to the membrane in a calcium-dependent manner, enabling the monitoring of sperm labeling and the detection of changes in fluidity [31,32]. The above corresponds with increased tyrosine phosphorylation, indicative of hyperactivated motility [33]. This assay is being applied for the first time in any non-avian reptile and represents a valuable reference for further studies on seminal parameters in other species. Incubation induced significant changes, with a decrease in the F pattern (non-capacitated sperm) over time and a higher percentage of the B pattern (capacitated sperm) after 2 h. Similar observations in dogs [34], mice [27], and boars [35] have been reported. The AR pattern increased from the second hour. These findings were confirmed by means of the lectin-binding assay, which specifically binds to spermatozoa with complete acrosome content [36]. Similar results were found in chickens under mammalian capacitating conditions [37]. Based on these findings, we inferred that sperm incubated under the described conditions underwent molecular changes consistent with capacitation, attributable to medium composition.

The constituents of the BWW medium induce diverse changes in the sperm. Albumin, for instance, modifies lipid composition and membrane fluidity by reducing plasma membrane cholesterol content [26,28]. Moreover, the presence of bicarbonate (above 15mM) in the medium initiates early changes that promote sperm capacitation by activating adenylate cyclase and elevating intracellular cAMP levels, resulting in the hyperpolarization of the plasma membrane and increased intracellular pH [2,38]. Also, calcium ions play a crucial role in this process by activating protein kinase A (PKA), which phosphorylates proteins involved in sperm functions like hyperactivation and the acrosome reaction [39,40]. Although the effects of glucose are unknown, it is essential for capacitation in mice, while pyruvate and lactate may inhibit it [27]. 

The incubation time has recently been recognized as a significant factor affecting sperm quality. In vitro studies on human sperm have shown a wide range from 1 to 24 h to capacitation induction, leading to sperm subpopulations with varying degrees of functionality [24]. We found the changes in sperm assessments starting at two hours, which accentuated at the third hour, accompanied by a significant decrease in total motility. However, the limited sperm volumes in lizards hamper our ability to incubate for longer periods or devise protocols to select the sperm with capacitation-associated effects.

Further research on changes in oviductal content and functional assays in both mated and unmated females would offer valuable insights [41] for optimizing the formulation of a more suitable medium. Incubation with calcium or progesterone ionophores can also be investigated, as both activate PKA-mediated signaling pathways, potentially improving efficiency in capacitation induction [39]. 

## 5. Conclusions

Our study revealed some suggestive changes associated with sperm capacitation, such as a change in the type of movement characterized by increased and deeper flagellar beat amplitude, an increased occurrence of capacitation patterns, and damaged sperm in the acrosome after two hours of incubation. These observations support the idea that this process also occurs in saurian. While sperm analysis is a valuable method for assessing certain functional changes, there are additional approaches required to validate this process. Establishing if sperm capacitation is a prerequisite for acquiring fertilization competence is crucial to improving the success of the implementation of any ART in this group of animals.

## Figures and Tables

**Table 1:** Morphometric and semen characteristics of male lizards *(Sceloporus torquatus)*.

Body Weight (g)	Snout–Vent Length (cm)	Vent–Tail Length (cm)	Number of Ejaculates	Total Semen Volume (μL)	Sperm Concentration (×10^6^/mL)
28.66 ± 4.2 (12.7–52.1)	8.73 ± 0.5 (6.0–11.5)	8.84 ± 0.6 (4.7–11.0)	2.00 ± 0.4 (1.0–3.0)	3.21 ± 1.31 (2.0–6.0)	94.23 ± 19.2 (18.3–220.0)

The data are the mean ± standard error of the mean; the range is shown in parentheses.

## Data Availability

Dataset available on request from the authors.
